# Predictors of Pharmaco-Resistance in Patients with Post-Stroke Epilepsy

**DOI:** 10.3390/brainsci11040418

**Published:** 2021-03-26

**Authors:** Simona Lattanzi, Claudia Rinaldi, Claudia Cagnetti, Nicoletta Foschi, Davide Norata, Serena Broggi, Chiara Rocchi, Mauro Silvestrini

**Affiliations:** Neurological Clinic, Department of Experimental and Clinical Medicine, Marche Polytechnic University, 60020 Ancona, Italy; claudiarinaldi16@gmail.com (C.R.); claudiacagnetti@libero.it (C.C.); nicoletta.foschi@ospedaliriuniti.marche.it (N.F.); dav.norata@gmail.com (D.N.); serena.broggi@gmail.com (S.B.); chiararocchi@hotmail.it (C.R.); m.silvestrini@univpm.it (M.S.)

**Keywords:** brain infarct, cerebral hemorrhage, stroke

## Abstract

Objectives: The study aimed to explore the clinical predictors of pharmaco-resistance in patients with post-stroke epilepsy (PSE). Methods: Patients with epilepsy secondary to cerebral infarct or spontaneous intracerebral hemorrhage were included. The study outcome was the occurrence of pharmaco-resistance defined as the failure of adequate trials of two tolerated and appropriately chosen and used antiseizure medication schedules, whether as monotherapies or in combination, to achieve sustained seizure freedom. Results: One-hundred and fifty-nine patients with PSE and a median follow-up of 5 (3–9) years were included. The mean age of the patients at stroke onset was 56.7 (14.9) years, and 104 (65.4%) were males. In the study cohort, 29 participants were pharmaco-resistant. Age at stroke onset [odds ratio (OR) 0.97, 95% confidence interval (CI) 0.93–0.99; *p* = 0.044], history of intracerebral hemorrhage (OR 2.95, 95% CI 1.06–8.24; *p* = 0.039), severe stroke (OR 5.43, 95% CI 1.82–16.16; *p* = 0.002), status epilepticus as initial presentation of PSE (OR 7.90, 1.66–37.55; *p* = 0.009), and focal to bilateral tonic-clonic seizures (OR 3.19, 95% CI 1.16–8.79; *p* = 0.025) were independent predictors of treatment refractoriness. Conclusions: Pharmaco-resistance developed in approximately 20% of patients with PSE and was associated with younger age at stroke onset, stroke type and severity, status epilepticus occurrence, and seizure types.

## 1. Introduction

Stroke is a leading disease worldwide, with an estimated incidence of 15 million cases annually [1]. Advances in stroke treatment have significantly reduced the overall mortality rate, whereas the number of survivors with complications and disability has increased [2,3,4]. Post-stroke epilepsy (PSE) is an important sequela of stroke, and represents one of the most common causes of acquired epilepsy. Cerebrovascular disease accounts for about 15% of all newly diagnosed epilepsy cases and nearly 50% among patients above 60 years of age [5,6,7]. With the world population aging and the increased prevalence of post-stroke survivors, the number of patients with PSE is expected to increase [2]. To date, little evidence exists about the prognosis of this condition, and additional information is needed for the proper counselling of patients and informed treatment decisions.

The aim of this study was to identify the clinical predictors of pharmaco-resistance in patients with PSE treated with antiseizure medications (ASMs).

## 2. Materials and Methods

### 2.1. Participants and Study Outcome

We retrospectively identified patients ≥16 years old who were referred to the Epilepsy Center of the Marche Polytechnic University, Ancona, Italy with a diagnosis of epilepsy secondary to cerebral infarct or spontaneous intracerebral hemorrhage and no history of seizures before stroke. According to the International League Against Epilepsy definition, seizures occurring within 7 days of the onset of stroke were classified as acute symptomatic seizures, seizures occurring beyond 7 days of the onset of stroke were classified as unprovoked seizures, and PSE was diagnosed as the occurrence of one or more unprovoked seizures [8]. Patients with follow-up <12 months were excluded from the analysis. Data on demographics, clinical history, medications, and seizure occurrence were collected from patients’ records of clinical visits. The study outcome was the occurrence of pharmaco-resistance: drug-resistant epilepsy was defined as the failure of adequate trials of two tolerated and appropriately chosen and used ASM schedules (whether as monotherapies or in combination) to achieve sustained seizure freedom [9].

### 2.2. Statistical Analysis

Values were presented as mean (standard deviation) or median (interquartile range) for continuous variables and as the number (percent) of patients for categorical variables. Comparisons were made through the Student’s *t* test, Mann–Whitney test or *χ*^2^ test, as appropriate. Simple and multivariable logistic regression analyses were performed to identify the predictors of pharmaco-resistance. For multivariate analysis, sex, age at stroke onset, stroke type, occurrence of acute symptomatic post-stroke seizures, seizure type, and any variables with *p* values < 0.02 from comparisons of baseline characteristics were identified for statistical adjustment. Results were reported as odds ratio (OR) with associated 95% confidence interval (CI). Results were considered significant for *p* values < 0.05 (two sided). Data analysis was performed using STATA/IC 13.1 statistical package (StataCorp LP, College Station, TX, USA).

## 3. Results

Of 172 patients with PSE initially identified, 13 patients were excluded due to follow-up <12 months. Accordingly, 159 patients with PSE and a median follow-up of 5 (3–9) years were included in the analysis. The mean age of the patients at stroke onset was 56.7 (14.9) years, and 104 (65.4%) were males. Cerebral infarct occurred in 102 (64.2%) and cerebral hemorrhage in 57 (35.8%) patients. Blood pressure lowering agents were taken by 121 (76.1%), antidiabetic drugs by 30 (18.9%), and statins by 49 (30.8%) patients.

In the study cohort, 29 (18.2%) participants were pharmaco-resistant. Drug-resistance occurred in 13 out of 102 (12.8%) patients with ischemic and 16 out of 57 (28.1%) patients with hemorrhagic stroke. Baseline characteristics of the patients according to treatment responsiveness are shown in Table 1. Patients refractory to treatment presented more frequently intracerebral hemorrhage (*p* = 0.016) and severe stroke (*p* = 0.001) in comparison to responsive patients. Status epilepticus was the first epileptic symptom in 5 (3.9%) responsive and 7 (24.1%) refractory patients (*p* < 0.001), and focal to bilateral tonic seizures were more commonly observed among patients with drug-resistant epilepsy (*p* = 0.013).

According to multiple regression analysis, age at stroke onset, stroke type and severity, status epilepticus as the first epileptic symptom, and seizure type were independent predictors of pharmaco-resistance: patients with younger age at stroke onset (OR 0.97, 95% CI 0.93–0.99; *p* = 0.044), history of intracerebral hemorrhage (OR 2.95, 95% CI 1.06–8.24; *p* = 0.039), severe stroke (OR 5.43, 95% CI 1.82–16.16; *p* = 0.002), status epilepticus as initial presentation of PSE (OR 7.90, 1.66–37.55; *p* = 0.009), and focal to bilateral tonic-clonic seizures (OR 3.19, 95% CI 1.16–8.79; *p* = 0.025) had a higher likelihood of being refractory to ASM treatment (Table 2).

## 4. Discussion

Pharmaco-resistance developed in approximately 20% of the patients with PSE included in this study and was associated with younger age at stroke onset, intra-cerebral hemorrhage, severe stroke, occurrence of status epilepticus as the first late-onset epileptic symptom, and focal to bilateral tonic-clonic seizures.

Around one third of the patients with epilepsy in general cannot achieve seizure control, despite the increasing availability of therapeutic options. Importantly, not all epileptic patients carry the same risk to be refractory to ASMs, and etiology can play a role. Despite differences in study cohorts, definitions, and follow-up times, the rate of treatment refractoriness we found is consistent with the figures reported in prior studies and suggests that, although PSE has overall a good prognosis and patients are generally responsive to pharmacological treatment, a not negligible percentage of cases become drug resistant [10,11,12,13,14].

Thus far, many investigations have shed light on the risk of seizures after stroke, and predicting tools have been developed for the early identification of patients at a higher risk of developing PSE. Conversely, the factors that can predict intractable PSE have been largely ignored.

Pharmaco-resistance in epilepsy is likely to be a multifactorial phenomenon that may incorporate both disease- and patient-related variables.

Alterations in the properties of ASM targets, including compositional changes in voltage-gated ion channels and neurotransmitter receptors, and the overexpression of efflux transporters at the level of the blood–brain barrier (BBB) can favor refractoriness by decreasing drug sensitivity and cerebral drug uptake [15]. Remarkably, widespread and long-lasting changes in the distribution of GABA_A_-receptor subunits and functional alterations in uninjured tissue surrounding brain lesion, as well as in remote cerebral regions, have been shown in experimental models [16,17]. The inflammatory response following brain injury can alter endothelial cell function and trigger profound changes in the expression and activity of ATP-binding cassette (ABC) efflux transporters at the BBB [18]. Induction of P-gp was found in newly generated capillaries after stroke [19,20,21], and breast cancer resistance protein (BCRP), which was not observed in the neurons of normal subjects and epileptic patients [22], has been detected in the neurons of stroke and refractory epileptic patients [20,23]. Sprouting of the synaptic connections between surviving deafferented neurons and the substantial remodeling of spared areas and pathways are crucial for recovery from brain damage. Nonetheless, post-stroke plasticity can also result in aberrant synaptogenesis [24]. The maladaptive organization of neuronal networks may suppress the endogenous seizure control system, generate synchronized neuronal circuits, and restrict ASMs from accessing neuronal targets, therefore leading to pharmaco-resistance [15]. Overall, it is conceivable that the presence of blood products in brain parenchyma and the extension of injury can act as crucial modifiers of the epileptogenic processes and trigger, favor, or amplify those aberrant responses that may contribute to developing seizures more refractory to therapy [25].

Common neurobiological factors can contribute to both epilepsy severity and pharmaco-resistance, and refractoriness can be inherent to disease severity [26]. In this regard, status epilepticus could result from reduced inhibition and enhanced excitability and be the epiphenomenon of pathophysiological changes characterizing refractory epilepsy. At the same time, there is also ample evidence that modifications triggered by status epilepticus itself, such as BBB disruption, inflammation, angiogenesis, and reactive synaptogenesis, play an important role in the reorganization of neuronal networks and the progression of seizure activity [27,28]. The present findings reinforced the association between status epilepticus occurrence and seizure intractability that has been observed in few prior studies of epilepsy in children and adults [29,30,31,32].

The predominant seizure type in patients with PSE was focal. Focal to bilateral tonic-clonic seizures were found in approximately one third of the cases, and associated with a higher risk of drug-refractoriness. In elderly patients with newly diagnosed epilepsy, seizure freedom was less commonly achieved by patients presenting with seizures evolving to bilateral convulsive than in patients with focal or generalized seizures [33,34]. A meta-analysis and indirect comparisons of responder rates demonstrated a trend toward a lower efficacy over secondary generalized tonic-clonic seizures than over all seizure types for the included ASMs, and only three drugs showed greater reduction of secondary generalized tonic-clonic seizures frequency than placebo [35]. As seizure initiation and propagation are triggered by different alterations of synaptic transmission [36], and secondary generalization reflects the involvement of specific cellular mechanisms and cortical and subcortical networks [37], ASMs might have different and lower efficacy in the prevention of focal to bilateral seizures in focal epilepsy.

Age at stroke onset was marginally associated with the risk of pharmaco-resistant PSE, with older age being a protective factor. Decreasing neuronal plasticity with age could smooth epileptogenicity and result in a reduced risk of pharmacoresistant epilepsy following stroke. This explanation has been previously advanced to explain the greater response to treatment observed among older adults [38]. Consistent with this finding, responding patients with PSE following cerebral hemorrhage were significantly older than resistant patients [39], and older age reduced the risk of stroke-related refractory epilepsy in a population-based sample of older adult stroke survivors [10].

A not significant higher prevalence of acute symptomatic post-stroke seizures was found among drug-resistant patients. Seizures occurring early after stroke are thought to be a reaction of neuronal cells to acute cerebrovascular injury, and reflect transient cellular biochemical dysfunctions, including variations in calcium and sodium concentrations, glutamate release, and the breakdown of membrane phospholipids. Acute symptomatic seizures have also, however, been associated with an increased risk of late unprovoked seizures, indicating that they might be a hallmark of epileptogenesis [40]. Our findings support the perspective of acute symptomatic post-stroke seizures as a consequence of temporary, acute, local metabolic disturbance rather than structural changes of neuronal networks and, although they may have role in the process of epileptogenesis after stroke, they do not overall seem to have a major influence on the development of treatment refractoriness. Of note, one single study identified the occurrence of acute symptomatic seizures as a predictor of drug-resistant epilepsy after cerebral hemorrhage [39]. Further studies are needed to definitively establish if a detrimental role of acute symptomatic seizures exists on the risk of pharmacoresistant PSE and whether this relationship varies according to stroke subtype.

We did not find any relationship of sex with drug refractoriness. To date, conflicting evidence exists about the predictive role of sex. Sex did not emerge as a significant predictor of refractory epilepsy in two studies recruiting participants with epilepsy of any cause of all ages [41] and older adults [33], whereas male sex was found protective in stroke patients aged 67 and over [10]. Although hormonal influence and metabolic changes act as potential biological modifiers, the differences across the studies did not allow for the comparison of results and the drawing of definitive conclusions. Given the decline in endogenous estrogen levels and the relative androgen excess during and after menopausal transition, further analyses in larger populations that may also explore the potential interaction between sex and age are warranted.

The current study has merit to provide evidence on the risk of drug-refractory PSE from the clinical perspective. Major strengths include the long-term follow-up of the patients and the real-world design, which allowed the generalizability of the findings to everyday clinical practice. Nonetheless, many shortcomings need to be considered. The retrospective analysis of data is prone to selection bias and misdiagnosis can occur even when medical reports are collected extensively. The inclusion of patients referred to an epilepsy clinic, which may represent a group preselected for age and severity, could limit the representativeness and generalizability of the findings to the general PSE population. Further, no information was available about the comorbidities and medications taken prior to stroke and during the acute phase, as well as whether vascular risk factors were adequately controlled after stroke. Therefore, we could not explore whether agents that have been suggested to reduce the risk of PSE may also influence the development of pharmaco-resistance [42]. The unavailability of genetic make-up data prevented us from exploring the contribution of genetic background to the development of pharmaco-resistance. The observational naturalistic design is, hence, able to develop preliminary insights and suggest working hypotheses. Prospective studies in larger cohorts of patients alongside molecular, neurophysiology, and neuroimaging analyses are warranted to validate the findings, identify additional predictors, and clarify the pathophysiological mechanisms underlying drug-refractoriness in PSE.

## 5. Conclusions

The available data on risks of acquiring PSE are not matched by knowledge on the condition itself and its prognosis, and there remains a large gap in the understanding of the causes and mechanisms of drug-refractoriness. The expected disease course is a reasonable question posed by patients and physicians. The identification of factors that may predict treatment response holds important practical implications to guide clinicians once a diagnosis has been made, and inform the health care system on the resources required when recovery from stroke is complicated by recurrent seizures.

## Figures and Tables

**Table 1:** Characteristics of patients according to treatment responsiveness.

	Responsive (*n* = 130)	Refractory (*n* = 29)	*p* Value
Male sex	86 (66.2)	18 (62.1)	0.676
Age at stroke onset	57.8 (14.7)	52.1 (15.3)	0.065
Familiar history of seizures	4 (3.1)	2 (6.9)	0.329
Hypertension	90 (69.2)	18 (62.1)	0.455
Diabetes mellitus	24 (18.5)	6 (20.7)	0.782
Dyslipidemia	51 (39.2)	11 (37.9)	0.897
Atrial fibrillation	19 (14.6)	2 (6.9)	0.267
Coronary heart disease	26 (20.0)	4 (13.8)	0.440
Stroke type			0.016
Cerebral infarct	89 (68.5)	13 (44.8)	
Intra-cerebral hemorrhage	41 (31.5)	16 (55.2)	
^a^ Stroke severity			0.001
Mild to moderate	75 (57.7)	7 (24.1)	
Severe	55 (42.3)	22 (75.9)	
Cortical involvement	87 (68.5)	24 (82.8)	0.126
^b^ Acute symptomatic post-stroke seizures	16 (12.3)	6 (20.7)	0.237
Status epilepticus at epilepsy onset	5 (3.9)	7 (24.1)	<0.001
Seizure types			0.013
Focal onset	67 (51.5)	11 (37.9)	
Focal to bilateral tonic-clonic	41 (31.6)	17 (58.6)	
Generalized/unknown onset	22 (16.9)	1 (3.5)	
Follow-up time	5 (3–9)	6 (3–12)	0.226

Data are mean (SD) or median (IQR) for continuous variables, and n (%) for categorical variables. ^a^ Stroke was defined as mild to moderate if NIHSS score at stroke onset was <16 and severe if initial NIHSS score was ≥16. ^b^ Seizures occurring within 7 days of stroke onset. Abbreviations: IQR = interquartile range, NIHSS = National Institutes of Health Stroke Scale, SD = standard deviation.

**Table 2:** Association between baseline characteristics and pharmaco-resistance.

Dependent Variable	* Adjusted OR (95% CI)	*p* Value
Male sex	1.04 (0.36–3.01)	0.948
Age at stroke onset	0.97 (0.93–0.99)	0.044
Cerebral hemorrhage	2.95 (1.06–8.24)	0.039
Severe stroke	5.43 (1.82–16.16)	0.002
Acute symptomatic post-stroke seizures	1.75 (0.53–5.76)	0.355
Status epilepticus at epilepsy onset	7.90 (1.66–37.55)	0.009
^‡^ Type of seizures		
Focal to bilateral tonic-clonic	3.19 (1.16–8.79)	0.025
Generalized/unknown onset	0.22 (0.02–2.12)	0.190

Values are from logistic regression models. * Adjustment for sex, age at stroke onset, stroke type and severity, occurrence of acute symptomatic post-stroke seizures, status epilepticus at epilepsy onset, and seizure types. ^‡^ Focal onset seizures as reference. Abbreviations: CI = confidence interval, NIHSS = National Institutes of Health Stroke Scale, OR = odds ratio.

## Data Availability

Anonymized data will be shared by request from any qualified investigator.
